# Mobile Phone Use during Gait: The Role of Perceived Prioritization and Executive Control

**DOI:** 10.3390/ijerph18168637

**Published:** 2021-08-16

**Authors:** Tal Krasovsky, Joel Lanir, Yasmin Felberbaum, Rachel Kizony

**Affiliations:** 1 Department of Physical Therapy, University of Haifa, Haifa 3498838, Israel; 2 Pediatric Rehabilitation Department, Sheba Medical Center, Ramat Gan 5262000, Israel; 3 Department of Information Systems, University of Haifa, Haifa 3498838, Israel; ylanir@is.haifa.ac.il (J.L.); yfelberb@is.haifa.ac.il (Y.F.); 4 Department of Occupational Therapy, University of Haifa, Haifa 3498838, Israel; rkizony@univ.haifa.ac.il; 5 Department of Occupational Therapy, Sheba Medical Center, Ramat Gan 5262000, Israel

**Keywords:** texting, walking, dual-task, cognitive flexibility, mental workload, gait stability

## Abstract

(1) Background: Mobile phone use during gait is associated with adverse health outcomes, namely increased risk of pedestrian injury. Healthy individuals can voluntarily prioritize concurrent task performance, but the factors underlying the impact of phone use during walking remain largely unknown. Thus, the objective of this work was to evaluate the relationship between subjective (perceived) prioritization, cognitive flexibility and dual-task performance when using a mobile phone during walking. (2) Methods: Thirty young participants walked for one minute with and without reading or texting on a mobile phone, as well as reading or texting while sitting. Walking performance (kinematics) was recorded, as well as phone use (text comprehension, text read/written), mental workload, perceived prioritization (visual analog scale), and cognitive flexibility (trail-making test). (3) Results: Texting while walking was associated with larger decreases in gait speed, larger gait variability, higher mental workload, and lower text comprehension compared to reading. Perceived prioritization was associated with walking dual-task costs (DTCs) (r = 0.39–0.42, *p* < 0.04) when texting, and better cognitive flexibility was associated with lower gait DTCs when texting (r = 0.55, *p* = 0.002) but not reading. (4) Conclusions: The context-dependent link between perceived prioritization, cognitive flexibility, and walking DTCs promotes our understanding of the factors underlying texting-while-walking performance. This could identify individuals who are more prone to dual-task interference in this increasingly common and dangerous task.

## 1. Introduction

Recent technological developments, specifically the proliferation of mobile phone use among people of all ages, have created an increasing competition for the attentional resources of individuals while walking [1,2]. Indeed, walking while using a mobile phone has been associated with a dramatic increase in pedestrian injury [3,4,5]. A myriad of factors may be associated with the impact of mobile phones on walking performance. In general, the decrease in performance of one task when adding another (dual-task cost (DTC)) is affected by the interplay between external factors such as the task [6,7], the environment [8], as well as internal factors associated with individual cognitive and motor capacity [9,10]. When texting and walking, people may be exceptionally vulnerable to cognitive-motor interference due to the fact that both texting and walking require similar resources (e.g., vision) [11]. As is the case with other activities concurrent with walking (i.e., performed simultaneously) [9], using a mobile phone while walking decreases the performance of both texting and walking. These decreases in performance may affect the stability of gait (e.g., increased gait variability during dual-tasking) [12]. Research is now beginning to explore the factors underlying these decreases, specifically the degree to which the level of performance depends on the availability of executive control (e.g., the ability to allocate attentional resources to several tasks [10]) resources. We previously found that lower DTC of walking in young healthy adults using a mobile phone is associated with better cognitive flexibility [12], and more recently, Stöckel and Mau-Moeller found that lower DTCs of walking during playing a game on the smartphone are associated with response inhibition, set shifting, working memory, and mental representation of walking [13]. In young healthy individuals, these relationships are identified when walking both with and without an obstacle [13] and in both quiet and busy environments [12], suggesting that higher DTCs represent reduced cognitive resources, even in young healthy individuals, across various environmental conditions. These factors may further underlie the adverse effects of walking with a mobile phone, such as pedestrian injury. However, the relationship between cognitive function and texting-while-walking performance may vary with the specific task, which may be critical for specific uses of mobile phones but not others. This is an important focus for research even for people with no limitations in cognitive flexibility (e.g., healthy young adults) because pedestrian injury rates for young adults using mobile phones [4] are much higher than those of older adults, potentially since they tend to use their phone more often when walking and/or demonstrate more risk-taking behaviors [5].

It is typically assumed that young healthy individuals with no deficits in executive control (e.g., due to learning disability or neurodevelopmental disorder) can prioritize, intentionally or unintentionally, one of the tasks (walking or phone use) to avoid injury. Yogev-Seligmann et al. have described dual-task (DT) prioritization during walking in the following way:

Task prioritization during walking involves the weighting of the motor and cognitive state during the specific dual-task situation, the functional reserve and compensatory capabilities of both modalities, and other related individual background features, such as personality, affect, and the expertise that is brought to bear [14].

According to the multiple resource approach to dual-task performance [15], in order to indicate that two concurrent tasks overlap in their demand of processing resources, three requirements need to be met: first, the decrease in performance of the two tasks needs to be demonstrated under DT conditions. Second, the performance of the two tasks should be flexibly manipulated according to prioritization; third, the effect of prioritization on performance needs to be larger when the task difficulty is higher [16]. For mobile-phone use (texting) during walking, the first two requirements have been demonstrated. Specifically, it was shown that both texting and walking performances are affected in DT conditions (for a review, see [11]) and by explicit prioritization instructions [8], such that when asked, people can focus on one task or the other, thereby altering the performance of both. However, the ability to perform this change of focus, which may be critical to ensure safe gait or accurate texting, may depend on task difficulty. It is currently unknown whether and how task difficulty modulates the relationship between prioritization and performance.

Typically, prioritization is measured by comparing performance in the two concurrent tasks, assuming that better performance implies higher prioritization. However, objective measures of performance may not fully capture prioritization. Perceived prioritization, i.e., the subjective perception of the importance of one task over the other in a DT situation, may better reflect implicit prioritization and vary with respect to objective performance. Individual characteristics, such as level of experience and skill, mood, or personality, can affect the attentional resources required for performance in DT conditions [14]. That is, perceived prioritization of one of the tasks may vary between individuals, whereas their objective performance may be similar. In a previous study, we demonstrated that perceived prioritization of texting was unrelated to DTCs for both young and older individuals [17]. However, that study involved a simple texting task, and it may be that the relationship between perceived prioritization and objective performance depends on task difficulty [9,14].

Thus, in the current study, we varied task difficulty by dissociating the visual-motor component of texting (requiring fine motor skill and scanning the keyboard) from the cognitive and visual components of reading (scanning the text). We compared reading and texting variants while walking and measured concurrent (DT) and separate (single-task (ST)) performance of all components: walking (gait speed and variability) and texting or reading (length of text written/read, comprehension). The main objectives of the study were (1) to compare dual-task performance and cost between walking while reading and walking while texting, and (2) to evaluate the relationship between perceived task prioritization, executive control, and dual task performance and cost. We hypothesized that DTCs and gait variability would be larger for texting compared to reading. Furthermore, since texting was expected to require more attentional resources than reading, it was hypothesized that perceived prioritization and executive control would be associated with DTCs when texting but not reading.

## 2. Materials and Methods

### 2.1. Participants

Thirty healthy young individuals (age 18–40 years old, 50% women) were recruited as a convenience sample using advertisements within the university. Participants were asked to have corrected to normal vision, be native Hebrew speakers, and able to read and write while walking (i.e., no dizziness). All participants owned and used a smartphone for at least one year. Participants were excluded if they had any orthopedic or neurological impairment affecting locomotion, or if they had pain during walking. All participants signed an informed consent form according to the university’s ethics committee guidelines (approval number 335/15).

### 2.2. Experimental Procedure

Participants arrived for a single session of ~1 h. Participants were asked to complete a demographic questionnaire, including details of smartphone usage (e.g., no. of texts sent per day). During testing, participants were required to walk for one minute while reading a paragraph of text or writing a paragraph of text on a mobile phone. No specific instructions regarding task prioritization were provided to participants in order to investigate implicit prioritization strategies. Walking trials were also performed without reading or texting (holding the phone in hand), and reading and texting were also performed while sitting. Experimental walking trials were performed along a quiet university corridor (30 m). A Nexus 5 mobile phone (LG electronics, Seoul, South Korea) was used for reading trials (dimensions 137.9 × 69.2 × 8.6 mm, weight 130 g) and a Samsung Galaxy S4 mobile phone was used for texting trials (dimensions 136.6 × 69.8 × 7.9 mm, 130 g). Custom-written Android applications were used for the reading and texting tasks. For both tasks, we used texts that were labelled as a 5th grade level of difficulty by the Ministry of Education and divided them into segments of 20 words shown per screen. For the texting task, the subject was required to copy the words that appeared on screen (20 words on each screen). When complete, the next 20 word paragraph appeared. At the end of each trial, the application automatically generated a file containing a detailed description of the number of letters typed. For the reading task, 20 words appeared on screen, and after reading them, the subject was required to tap the screen to bring up the next 20 words. Participants were allowed to practice the texting and reading tasks until they felt comfortable with them. To measure comprehension the text, four multiple choice comprehension questions were presented following each trial. One of the multiple-choice answers indicated “I did not read this part”. Participants were told beforehand that comprehension questions would be asked.

### 2.3. Measures

During walking trials, movement kinematics were collected using inertial movement sensors (Mobility Lab, APDM Inc., Portland, OR, USA): one placed on each ankle and one on the waist (48.5 × 36.5 × 13.5 mm, weight 22 g). Prioritization was measured using a visual analog scale administered at the end of the session for each dual-task separately. On a 10 cm line, participants were asked to which task they paid more attention (with one end being walking, and the other being the secondary task of reading or texting). In addition, subjects completed a subjective workload scale (NASA-Task Load Index [18]), rating the subjective level of task load in different domains (mental, physical, and temporal) as well as performance, effort, and frustration. These scores were averaged, generating a raw score of task workload [19]. Participants also completed both parts of the trail-making test [20], which examines visual scanning, divided attention, and cognitive flexibility. The test measures the time required to draw a line connecting a series of characters, either numbers (TMTa) or alternating numbers and letters (i.e., 1-A-2-B-3-C… (TMTb)); the latter emphasizing cognitive flexibility [21]. The same researcher (YF) was present in all experimental sessions.

### 2.4. Data Analysis

Spatiotemporal gait kinematics (stride length and time) were calculated from motion sensor data using custom-written MATLAB code (Mathworks, Natick, MA, USA), similar to previous work [12]. Gait speed and the coefficient of variation (CV) for gait speed were computed. For both walking and phone-use variables (i.e., gait speed, stride length, stride time, and no. of words or letters read or written), dual task costs were calculated using the formula:

DTC (%) = 100 × [(DT performance − ST performance)/ST performance](1)

The number of words read during reading trials was obtained directly by counting the number of screens swapped during a trial and adding to it the number of words read on the last screen (according to the subject’s verbal account). The number of letters written during texting trials was obtained from the application log file. Since the outcome of the reading task (no. of words) differed from the outcome in the texting task (no. of letters), comparison was only performed for dual-task costs.

A comprehension score for the text to which the subject was exposed was calculated by dividing the number of correct answers each participant answered for each trial by the total number of questions that the subject answered (disregarding those in which they answered “I did not read this part”) and multiplying by 100. DTCs were not calculated for reading comprehension due to the limited number of response options.

Perceived task prioritization (visual analog scale) was converted to percentage (0% = walking priority, 100% = reading/texting priority).

### 2.5. Statistical Analysis

Statistical analysis was performed with SPSS (IBM SPSS Statistics for Windows, Version 25.0, IBM Corp., Armonk, NY, USA). Dual-task costs of walking (gait speed, stride length and stride time) and phone task (speed of reading or texting), as well as gait variability (gait speed (CV)), task workload, and text comprehension, were compared between reading and texting tasks using paired-sample Student’s *t*-test and Wilcoxon signed-rank test for non-normally distributed variables. Spearman’s rho test was used to correlate DTCs with task prioritization and with visual scanning and cognitive flexibility (TMTa and b), as well as with experience in texting (no. of texts sent per day). Effect sizes ($\eta^{2}$) for non-parametric tests were calculated according to Fritz et al. (2012). Sample size was determined using G*power 3.1.9.7 [22]. For Wilcoxon’s signed-rank test, to detect a medium effect size (d = 0.55) for the effect of task on a DTC with α = 0.05 and 80% power, *N* = 30 participants were required.

## 3. Results

Subject characteristics are detailed in Table 1. No age or sex differences were noted in task performance variables, and men and women were thus grouped. One participant was removed from the final sample due to technical problems with the measurement equipment. A trend (Z = −1.76, *p* = 0.08, $\eta^{2}$ = 0.1) was noted for prioritizing of texting compared to reading during dual-task walking trials (median (IQR): 81.1% (20.13) for texting and 74.8% (20.13) for reading). Accordingly, there was no difference in task workload for reading or texting while sitting, but when walking, texting was associated with a higher task workload than reading (Z = −2.55, *p* = 0.011, $\eta^{2}$ = 0.22). The number of texts per day sent by participants (Table 1) was negatively related to gait DTCs while reading (gait speed: r = −0.49, *p* = 0.006, stride length: r = −0.49, *p* = 0.006) and marginally related to gait DTCs while writing (gait speed: r = −0.35, *p* = 0.06, stride length: r = −0.36, *p* = 0.057, stride time: r = −0.36, *p* = 0.057), suggesting that people who had more experience with texting tended to have lower DTCs of walking, especially when performing the easier task. The number of texts per day was not associated with perceived task prioritization.

**Figure 1:**
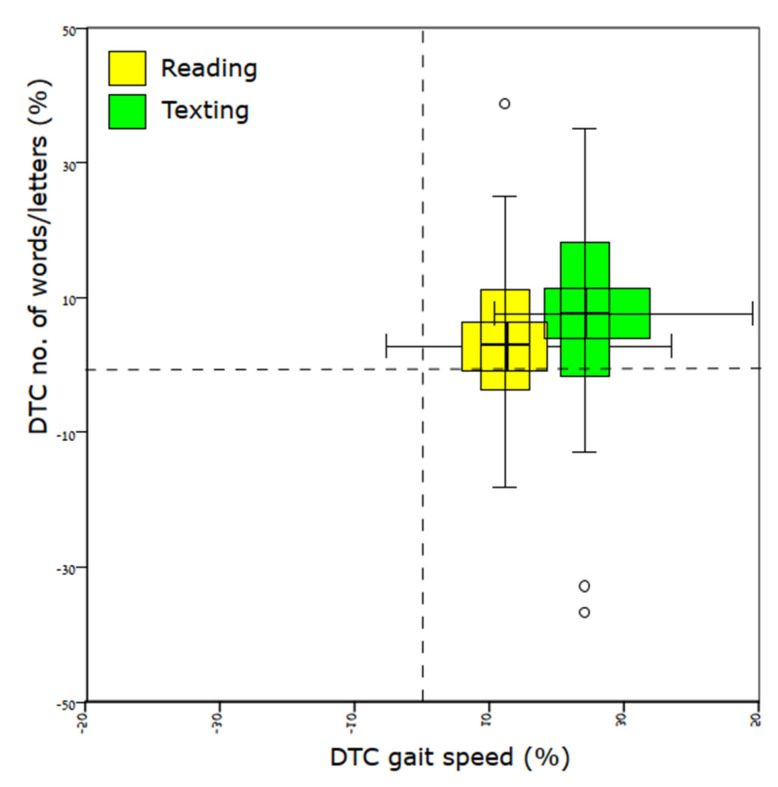
Box plots of dual-task costs (DTCs) for one aspect of performance of the mobile phone task (reading or texting speed) on the ordinate and performance of the walking task (gait speed) on the abscissa. Positive values denote worse performance for both tasks. Results indicate that texting (green) compared with reading (yellow) during walking incurs higher DTCs for both tasks.

Gait and reading/texting performance in each condition is presented in Table 2, and the DTCs for each condition are presented in Figure 1. As shown in Figure 1, for all tasks, DTCs were positive, indicating that performance decreased during dual-task conditions for all tasks. The DTCs of gait speed, stride time, and stride length were larger when texting than when reading (Z = −4.7, *p* < 0.001, $\eta^{2}$ = 0.76; Z = −4.7, *p* < 0.001, $\eta^{2}$ = 0.76; Z = −4.3, *p* < 0.001, $\eta^{2}$ = 0.64 respectively). Similarly, gait speed variability was higher when texting compared to reading (Z = −3.96, *p* < 0.001, $\eta^{2}$ = 0.54). However, DTCs of the length of text read or written did not vary between reading and texting conditions, suggesting a similar decrease in this aspect of phone usage for both tasks. Nevertheless, text comprehension was better when walking and reading compared to texting (Z = −2.71, *p* = 0.007, $\eta^{2}$ = 0.25), but was similar for both conditions (reading and texting) when sitting.

Perceived prioritization to texting was associated with a DTC of gait speed during texting (r = 0.39, *p* = 0.036) and a DTC of stride time during texting (r = 0.41, *p* = 0.028). A trend was also found for a relationship of DTC with stride length (r = 0.34, *p* = 0.07). Participants who prioritized texting showed more variability in walking speed (r = 0.50, *p* = 0.006). In contrast, in reading, perceived prioritization was not found to be associated with the DTCs of either gait speed, stride length, or stride time, nor with gait variability. For both reading and texting, perceived prioritization was not associated with a DTC of the amount of text read or written.

The DTC of walking performance (stride time) during texting was associated with TMTb (r = 0.55, *p* = 0.002) and a trend was identified for a relationship with TMTa (r = 0.36, *p* = 0.057), such that people with better TMT scores had lower costs for walking. In contrast, no relationship was found for DTCs of walking during reading with either TMTa or TMTb scores.

## 4. Discussion

This work examined the correlates of performance of two mobile phone tasks (reading and texting) performed while walking in young healthy adults. We confirmed that texting compared to reading during walking increased task workload and the DTC of gait and decreased stability (i.e., increased gait variability) as well as comprehension of the text. In both dual-task conditions (reading and texting) participants prioritized the mobile phone task. However, perceived prioritization and cognitive flexibility were associated with task performance only for the texting, but not for the reading dual-task. This work highlights the role of task complexity in connecting individual characteristics (cognitive flexibility and perceived prioritization) with dual-task performance, specifically for the increasingly popular task of texting while walking.

Our results are in agreement with the accumulating evidence for the decreases in walking associated with texting in young healthy adults (for a review, see [11]) as well as older populations [1]. Moreover, the current work confirms the previous results showing that texting is associated with increased walking DTC in comparison to reading or looking at the phone [7,23]. In the current work, we further show that texting caused a larger increase in gait variability, which may be associated with increased risk of falling [24]. Compared with falls of older adults [25], falls in young adults are typically understudied, their rates are high [26], and they may still pose a significant health risk. Thus, understanding the stability characteristics of texting while walking, which is an increasingly common task, is essential even in young, healthy adults. From a practical perspective, these results demonstrate the need to raise awareness of young adults to the stability risk associated with texting compared to reading while walking.

Importantly, the current work assessed the performance of the texting task as well, further demonstrating that texting during walking is associated with worse comprehension than reading during walking. Text comprehension is an ecologically valid measure of performance on the reading and texting task, which enables measurement of the amount of interference of walking for both tasks in terms of the ability to use the information read or typed. In contrast, the amount of text read or written decreased similarly when adding the secondary task (i.e., similar DTCs for text read or written), suggesting that walking similarly impaired reading and writing speed. This implies that the shared resources required to perform the texting task during walking limit some aspects of task performance more than others.

Recent studies have begun to explore the associations between specific cognitive functions and the DTCs of using a mobile phone while walking [13,27]. However, the specific cognitive processes that are important for this task are not agreed upon. Specifically, the results of the current study, suggesting that cognitive flexibility, as measured by TMTb, is associated with walking DTCs, are in accordance with those of Niederer et al. [27] and our previous work [12] but not with Stöckel and Mau-Moeller [13]. These discrepancies may be explained by the differences between tasks used in these studies. However, whereas the task used in the current work is different than the gaming task used by Stöckel and Mau-Moeller, the decreases in gait speed and variability are strikingly similar between studies (~25% and ~60%, respectively) suggesting that task difficulty (for texting) in the current work is comparable to that of the gaming task of Stöckel and Mau-Moeller [13]. Thus, it may not be task difficulty but rather the specific motor and cognitive requirements of the task that underlie these discrepancies. The origin of the relationship between walking DTCs and the cognitive flexibility identified here may be the increased need to shift gaze to the screen when texting [28]. Visual search was shown to affect postural control during standing [29]. It seems that the additional task component of locating the letters and checking written text quality may require significant additional visual resources and set-shifting in comparison to reading. However, the fact that the relationships identified in these studies (including the current one) are moderate at most suggests that the cognitive processes underlying texting while walking are multifactorial. Furthermore, the relationship identified between the amount of experience with texting and gait DTCs suggests that, to an extent and at least in young healthy individuals, gait DTCs can be modifiable with time and experience. Thus, individual characteristics, such as cognitive flexibility as well as life experience, should be taken into consideration when evaluating the personal level of risk of using mobile phones during walking.

Intact executive control enables an individual to dynamically change their dual-task prioritization strategies, usually achieved as an implicit process [16,30]. This work demonstrated that the association of prioritization with task performance is larger when task difficulty is higher (i.e., higher workload) [16]. This is in line with the requirements of the multiple-resource model of dual-task performance [15], suggesting that the two concurrent tasks (walking and mobile phone use) overlap in their demand for processing resources. The finding that the link between perceived prioritization and objective measures of task performance depends on task difficulty suggests that the accuracy of the subjective perception of users of mobile phones during walking is context-specific. This suggests that for increasingly complex tasks, healthy individuals should be aware that their intuitive prioritization is linked to altered gait performance. These findings further indicate the need to investigate perceived prioritization as a proxy for implicit prioritization in people with attentional or executive deficits. In a previous study, we showed that perceived prioritization mediated the age effect on the DTC [17].

This study has several limitations. First, we did not include an extensive cognitive profile for participants. Thus, further work may be required to map the specific cognitive functions relevant for mobile phone use while walking. However, use of the TMT is comparable to previous work [12,27] and supports the generalizability of results. An additional limitation of this work is that the distribution of the text on screen was not identical between tasks (i.e., text was smaller for texting) due to a need to also display the written text. This may have added to the visual complexity of the texting task. However, the number of words on screen was kept identical between tasks and the results showed that the decreases in amount of text read or written between single and dual tasks were similar between conditions.

## 5. Conclusions

The current work confirms that texting while walking results in higher gait DTCs compared to reading, as demonstrated, amongst others, by decreased gait stability and text comprehension. This further emphasizes the need to raise the awareness of young adults of the stability risk associated with texting compared to reading while walking. Moreover, this work demonstrates that the relationship of the DTCs of walking with perceived prioritization and executive control depends on task requirements and on the amount of shared resources between tasks. This finding in young, healthy adults promotes our understanding of the factors underlying the performance of this increasingly common dual task of texting while walking. Furthermore, it suggests that individual characteristics such as cognitive flexibility and life experience should be considered when evaluating the personal level of risk of mobile-phone use during walking. The ability to identify individuals who are more prone to the health risks associated with dual-task interference in the everyday task of texting while walking can promote safe ambulation in community settings. Future investigation is warranted in older adults and clinical populations in order to relate the motor and cognitive capacity of individuals to dual-task performance and assist health professionals in developing strategies, i.e., using perceived prioritization, to ensure safety during complex walking tasks.

## Figures and Tables

**Table 1:** Participants’ characteristics.

Variable	Value
Sex	15 F/14 M
Age (years)—Mean (SD)	26 (4.18)
Height (cm)—Mean (SD)	169.9 (10.5)
Years of study (years)—Mean (SD)	12.9 (4.2)
Dominance (left/right hand)	4 L/25 R
Current phone’s operating system	26 Android/3 iOS
Use of smartphone (% of subjects)	
Calls only	10.3
Calls and text messages	13.8
Calls, text messages, and Internet	44.8
Calls, text messages, Internet, and reading	31.3
Texts per day (% of subjects)	
Under 10	10.3
10–30	20.7
31–50	27.6
51–70	13.8
71–90	10.3
Above 90	17.2
Reading on a mobile while walking (% of subjects)	
Not at all	27.6
>25% of walk time	48.3
25–50% of walk time	3.4
50–75% of walk time	17.2
>75% of walk time	3.4
Writing on a mobile while walking (% of subjects)	
Not at all	3.4
>25% of walk time	72.4
25–50% of walk time	24.1
50–75% of walk time	0
>75% of walk time	0
Ever stumbled/fell while using phone (% of subjects)	
No	82.8
Yes, while talking	0
Yes, while writing	6.9
Yes, while reading	0
Yes, both writing and reading	10.3
If yes, how often (% of subjects)	
Rarely	75.0
Occasionally	25.0
Often	-
TMTa (seconds)—Mean (SD)	25.2 (8.36)
TMTb (seconds)—Mean (SD)	46.9 (12.1)

TMT = trail-making test.

**Table 2:** Outcomes for walking and reading or writing for single- and dual-task trials. ST = single task, DT = dual task.

Variable	ST Reading	ST Writing	ST Walking	DT Walking + Reading	DT Walking + Texting
Gait speed (m/s)			1.24 ± 0.24	1.08 ± 0.25	0.93 ± 0.23
Stride length (m)			1.28 ± 0.26	1.21 ± 0.23	1.10 ± 0.23
Stride time (s)			1.07 ± 0.07	1.11 ± 0.09	1.21 ± 0.13
Gait speed variability (COV)			0.048 ± 0.03	0.052 ± 0.032	0.076 ± 0.054
Words (for reading)/letters (for writing) read/written	207.3 ± 41.66	146.7 ± 34.3		193.6 ± 42.9	139.0 ± 39.6
Text comprehension (%)	77 ± 25	64 ±39		76 ± 24	53 ± 42
Task workload (RTLX 0–5)	3.29 ± 0.78	3.28 ± 1.06	3.22 ± 0.72	3.66 ± 1.04	4.00 ± 1.23

COV = coefficient of variation. RTLX = Raw NASA Task Load Index [18].

## Data Availability

The data presented in this study are available on request from the corresponding author. The data are not publicly available due to ethical approval constraints.
